# Breast Cancer Detection—A Synopsis of Conventional Modalities and the Potential Role of Microwave Imaging

**DOI:** 10.3390/diagnostics10020103

**Published:** 2020-02-14

**Authors:** Brian M. Moloney, Declan O’Loughlin, Sami Abd Elwahab, Michael J. Kerin

**Affiliations:** 1 Department of Diagnostic Radiology, Galway University Hospitals, Galway H91 YR71, Ireland; 2 Discipline of Surgery, Lambe Institute for Translational Research, School of Medicine, National University of Ireland Galway, Galway H91 YR71, Ireland; 3 Electrical and Electronic Engineering, National University of Ireland Galway, Galway H91 CF50, Ireland

**Keywords:** breast imaging, breast cancer, microwave breast imaging, multimodal imaging

## Abstract

Global statistics have demonstrated that breast cancer is the most frequently diagnosed invasive cancer and the leading cause of cancer death among female patients. Survival following a diagnosis of breast cancer is grossly determined by the stage of the disease at the time of initial diagnosis, highlighting the importance of early detection. Improving early diagnosis will require a multi-faceted approach to optimizing the use of currently available imaging modalities and investigating new methods of detection. The application of microwave technologies in medical diagnostics is an emerging field of research, with breast cancer detection seeing the most significant progress in the last twenty years. In this review, the application of current conventional imaging modalities is discussed, and recurrent shortcomings highlighted. Microwave imaging is rapid and inexpensive. If the preliminary results of its diagnostic capacity are substantiated, microwave technology may offer a non-ionizing, non-invasive, and painless adjunct or stand-alone modality that could possibly be implemented in routine diagnostic breast care.

## 1. Introduction

Breast cancer is the most commonly diagnosed female malignancy and is the fifth leading cause of cancer death. Annually, approximately 1.67 million cases of invasive breast cancer are diagnosed worldwide, and it is the cause of approximately 522,000 deaths [1]. The lifetime risk of developing breast cancer stands at one in eight females [2]. The incidence of breast cancer in developed countries continues to rise; however, the rate of mortality has undergone a substantial decline [3]. This is attributable to both improved methods of treatment, and implementation of screening programs and improved imaging techniques [4]. Critically, early detection ensures the best outcome for the patient, with survival grossly determined by the stage of the disease at the time of initial diagnosis [5]. In this era of a rising incidence of breast cancer, ensuring diagnosis at the earliest possible stage requires further improvement in the capabilities of current diagnostic modalities and the development of novel imaging systems. Here current conventional imaging applications are reviewed, such as mammography, digital breast tomosynthesis, ultrasonography, and magnetic resonance imaging. These modalities exploit a variety of properties of biological tissues to form an image:

- Mammography and digital breast tomosynthesis exploit the attenuation of x-rays as they pass through breast tissue measured by the attenuation coefficient of the tissue;
- Magnetic resonance imaging uses radio waves, magnetic field gradients, and contrast agents to excite and measure the location of hydrogen atoms;
- Ultrasound exploits differences in acoustic impedance between tissue types as sound waves propagate in the breast.

The potential of microwave breast imaging, a non-ionizing imaging modality that represents a promising method of breast cancer detection, is then discussed. Microwave breast imaging exploits the dielectric properties of biological tissues to form images, potentially providing complementary information to conventional modalities. While it is not expected that the acoustic, x-ray attenuation and dielectric properties are completely independent (all of the x-ray attenuation, acoustic impedance, and dielectric properties are influenced by water content), it is still expected that the dielectric properties would be influenced by other factors such as fractions of bound water and ion concentrations, suggesting that microwave breast imaging may be a useful adjunct imaging modality.

## 2. Mammography

Although the use of radiography to differentiate benign from cancerous specimen tissue was recorded as far back as 1913 [6], its use to clinically evaluate the symptomatic patient was delayed until the 1930′s, when Stafford Warren, who is credited with the invention of the mammogram, pioneered a stereoscopic technique for mammography [7]. To this day, mammography remains the gold standard investigation of symptomatic women aged 40 and over and for the whole population breast cancer screening. While it offers a cost-effective method of investigating breast cancer, its effectiveness is severely hindered by several limitations. For most women, breast compression is an uncomfortable experience. In a study of 954 patients, Keemers-Gels et al. [8] reported that as many as 79% of patients undergoing breast cancer screening found mammography to be mild to severely painful. Additionally, this study established that the pain associated with breast compression during mammography was the main deterrent for women who indicated that they would not attend for further screening. Mammography is also limited in identifying tumors that present without a characteristic mass (as is frequently the case with invasive lobular breast cancer), without calcification (as can occur in entities like non-calcified ductal carcinoma), and in breasts of higher density [9]. The sensitivity of screening mammography, which aims to detect pre-clinical breast cancer in asymptomatic women, is estimated to be between 68 and 90%, with the lower margin of this range applicable to mammographically dense breasts, as is common in younger aged women. Even in women with lower breast density (> 75 years), the sensitivity for screening mammography is 88.4%. With diagnostic mammography, which involves the evaluation of patients with symptoms and signs suggestive of breast cancer, the sensitivity is slightly higher, at 93% [10]. 

### 2.1. Screening Mammography

Uninterrupted controversy continues to surround screening mammography and the true impact it has on breast cancer. While early breast cancer detection is undoubtedly beneficial for the patient, the benefits of screening appear to be less than as first thought. On the one hand, the use of this modality in organized screening programs remains the only screening test proven to reduce breast cancer mortality supported by randomized trials and subsequent meta-analyses [11,12]. In the randomized control trials (RCTs) and meta-analysis (Table 1) conducted on screening mammography deemed to be of sound methodologic quality, there was a reduction in breast cancer mortality between 20% to 45% in female participants aged 40 to 70 years. Modern mammography screening cites a similar reduction in breast cancer mortality of 28% [13]. 

However, these RCTs have been subject to criticism, with concerns raised that they are irrelevant to the modern era of breast cancer management as they were conducted prior to the introduction of taxane adjuvant therapy and the standardized acceptance of adjuvant hormone treatment for estrogen receptor-positive disease [22]. This has led some authors to doubt that the benefits of screening mammography would persist under present conditions.

In a seminal publication in 2012, Bleyer et al. [23] interrogated the US Surveillance, Epidemiology, and End Results data to examine trends of early- and late-stage breast cancer among women 40 years of age or older over a 30-year period. In this study, the group demonstrated that screening mammography resulted in a doubling in the annual incidence of early-stage breast cancer that is detected. However, it was calculated that breast cancer was overdiagnosed in up to 31% of all cases, accounting for 1.3 million women over the past 30-year study period. Furthermore, this group demonstrated that screening mammography only marginally reduced the rate of incidence of late-stage breast cancer and concluded that screening was having a minor impact on breast cancer mortality overall. 

In a 2012 Cochrane review, Gøtzsche et al. [24] extracted data from eight RCTs, comprising 600,000 women (39–74 years) comparing mammographic screening with no mammographic screening. It was demonstrated that in trials with adequate randomization, screening did not impact breast cancer mortality after a 10 year follow up (Relative Risk 1.02, 95% CI 0.95 to 1.10). In 2013, the Swiss Medical Board (SMB) estimated the benefits, harms, and cost-effectiveness of organized breast screening. This report highlighted the difficulty in demonstrating that the benefits of mammography screening outweighed the harms. Accepting that screening offers a relative risk reduction of breast-cancer mortality of 20%, it was stressed that this came at the cost of a substantial diagnostic cascade. Contrary to public perception, for every female breast-cancer death prevented over a 10-year course of annual screening (initiated at 50 years), as many as 670 patients are likely to have a false-positive outcome with repeat mammography; as many as 100 will have an unnecessary biopsy, and up to 14 will have an overdiagnosed breast cancer that would never have presented clinically [25]. A conclusion was drawn that screening mammography was not cost-effective, and the group recommended that national screening be abolished [26]. To add to the murkiness of international opinion, a 2015 review funded by the Swiss cancer league was published that identified that the SMB report was fundamentally flawed in several aspects expected in appropriate cost-effectiveness analysis [27]. 

In the UK, following the introduction of the NHS Breast Cancer Screening Program in 1988, women aged 50 to 70 are invited to participate in breast screening every three years, with women over 70 able to self-refer. In 2012, an independent review was convened to reach conclusions about the benefits and harms of breast screening on the currently available literature. Focusing on the UK setting, the panel concluded that 20% was a reasonable estimate of the relative risk reduction of breast cancer mortality and that breast screening saves one life for every 2,000 women screened, or up to 1,300 lives per year in the UK [28].

In the US, a review of five Breast Cancer Surveillance Consortium breast imaging registries, consisting of over 400,000 patients aged 40–90, with linkages to pathology and tumor registries was published in February 2016 [29]. The rates of false-positive (a patient recalled for further investigation, with no underlying breast cancer) and false negative (a patient-reported as normal, despite underlying breast cancer) results and risk factors for recommendations for further imaging and biopsies were analyzed. Rates of false-positives were highest in women aged 40–49 (121.2 per 1000) and decreased with increasing age (50–59 (93.2 per 1000), 60–69 (80.8 per 1000)). This study highlighted that false-positive mammography results are more common in younger women and fortifies opinions that breast screening may be better suited for patients over the age of 50 years. This judgment was supported by the United States Preventive Services Task Force (USPSTF), who reviewed the evidence of breast cancer screening in reducing breast cancer-specific mortality, as well as treatment-related morbidity and harms of breast screening [30]. The USPSTF recommended biennial screening mammography for women between the ages of 50 and 74 years and recommended against routine screening mammography in women aged 40 to 49 years.

**Figure 1:**
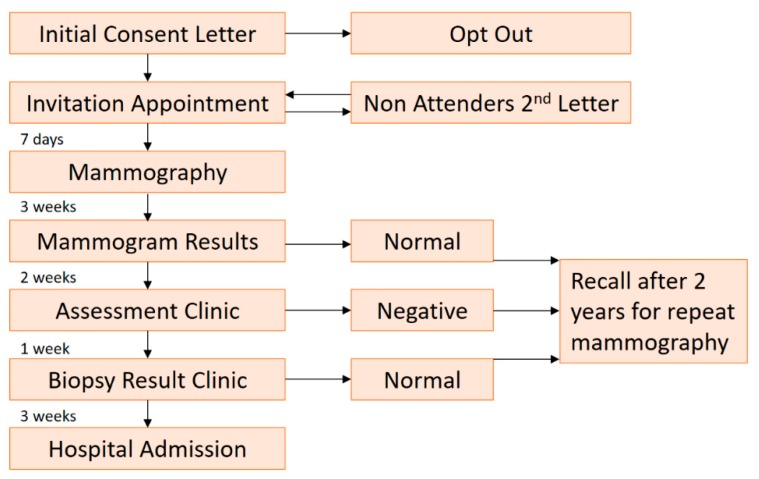
Screening process flow sheet and timescales for the BreastCheck screening program.

The Republic of Ireland National Breast Screening Program, BreastCheck, commenced in February 2000. The service offered a free digitalized screening mammography service to women aged 50 to 64 every two years in eleven of the 26 counties. The program was expanded to include a further three counties in 2004, and finally, to include all 26 counties in December 2007. The program is currently in the process of extending the age-range on a phased basis, with the intention that by 2021, all women aged 50–69 will be invited for screening [31]. Since its initiation, the target population invitation acceptance rate has remained somewhat constant over time ranging from 68% to 76% of those invited [31]. Two standard mammographic view are obtained, craniocaudal (CC) and mediolateral-oblique (MLO). The compression plane for CC is transaxial, while the MLO image plane is approximately 45 to 60 degrees from the axial plane, paralleling the course of the pectoralis muscle heading into the axilla. Each image is independently assessed by two consultant breast radiologists. Each breast radiologists must read at least 5,000 screening cases per year and have specific training in mammography. Patients who have suspicious abnormalities identified on their initial mammograms are recalled to a dedicated assessment clinic for further investigations, such as additional view mammography, ultrasound, or biopsy, as detailed in Figure 1. All cases requiring biopsy are discussed at a tertiary breast center multidisciplinary meeting [32].

### 2.2. Symptomatic Breast Mammography

Diagnostic mammography is indicated for women over the age of 35 years, presenting with a palpable lump. If a benign correlate for a palpable lump (i.e., fibroadenoma, lipoma, hamartoma, or benign lymph node) is distinguished on mammography, no further imaging or biopsy is required, and clinical follow-up is adequate. If no abnormality can be seen with mammography or a finding is distinguished that is not clearly benign, further imaging is usually deemed appropriate, with targeted breast sonography employed. In Ireland, National Quality Assurance Standards ensure that urgent referrals are seen within two weeks. These include (1) Patients over 35 years of age with a discrete lump (2) Patients with suspicious signs, including; ulceration, skin tethering, unilateral nipple eczema, recent nipple inversion retraction or distortion, or (3) Individuals that the General Practitioner deems to have a significant probability of breast cancer at any age, and finally (4) Patients who develop an acute breast abscess. Further referral pathways exist for routine referrals to be evaluated within 12 weeks [33].

## 3. Digital Breast Tomosynthesis (DBT) 

Tomosynthesis (3-Dimensional mammography) is a modification of digital mammography that uses a moving x-ray tube and digital detector to create a series of low dose exposures that are acquired from consecutive exposures along a limited arc angle while the breast is compressed. Individually, these low dose radiographs are only a fraction of the total dose used during conventional digital mammography. If a breast is imaged from 15 projections around a 45-degree arc, a radiograph will be generated every 3 degrees. These two-dimensional exposures are then used to reconstruct three-dimensional images of the breasts using algorithms to generate multiple, thin (usually 1 mm) slices in the same plane as the mammogram [34].

The addition of DBT to conventional digital mammography has been shown to increase rates of cancer detection. In a randomized clinical trial of women aged 45 to 75 years, cancer detection for patients screened with mammography and DBT was higher (*n* = 9777, 8.6 per 1000) than patients who underwent mammography alone (*n* = 9783, 4.5 per 1000, RR 1.9) [35]. This is counterbalanced by increased radiation dose, increased cost, and increased radiologist interpretation time [35]. Overall, the procedure is well-tolerated by patients, and its use is becoming increasingly widespread, usually at the discretion of the radiologist. 

## 4. Breast Sonography

Breast sonography offers a non-ionizing, high resolution, low cost, highly sensitive (81.7%) and specific (88%) instrument for investigating symptomatic patients [36]. Using a handheld transducer, breast sonography has a definitive role in establishing the relationships between glandular, fat, and fibrous components of breast tissue based on the different acoustic impedances of these tissues. This is difficult to elucidate by mammography alone, as the fibrous and glandular components of the breast have similar X-ray attenuation coefficients. While initially employed as a diagnostic adjunct to further assess mammographic findings and palpable abnormalities, technical advances, including improved spatial and contrast resolution, and higher-megahertz (MHz) transducers have facilitated the use of US to characterize solid masses and provide uncomplicated image guidance for needle biopsy [37]. 

Breast sonography is the modality of choice for further investigation of palpable breast findings that are not clearly benign and mammographic screen-detected abnormalities. Furthermore, breast sonography is advised as an initial investigation prior to mammography in women younger than 30 years or in female patients that are lactating or pregnant [38]. This is recommended both to prevent unnecessary radiation exposure to a population of patients who carry a low incidence of breast cancer [39] and, as women under 30 years of age, generally have a relatively higher breast tissue density [40], mammographic sensitivity is decreased [41]. 

## 5. Magnetic Resonance Imaging (MRI)

The role of MRI in breast cancer diagnosis and management continues to evolve. MRI, with the aid of contrast (Gadolinium) enhancement, offers high soft tissue distinction, multiplanar sectioning, and three-dimensional reconstructions of the breast. It affords a sensitivity (over 90%), which is more favorable than both mammography and breast sonography for breast cancer [42]. However, the specificity of lesion characterization with MRI remains low to moderate (approximately 72%) [43], rendering the distinction between cancer and benign pathology a challenge. 

The value of MRI as a screening adjunct in high-risk populations has been definitively recognized [44]. In the high-risk cohorts, such as patients with BRCA1 and BRCA2 deleterious mutations, TP53 mutations (Li–Fraumeni syndrome), or rare moderate-penetrance alleles such as CHEK2, ATM, and BRIP1, breast cancer can occur at a younger age, when breast density is higher. In these cases, tumors are often of a high grade and present at an advanced clinical stage [45]. In a comprehensive, multicenter randomized control trial of 2,809 high-risk women with dense breasts, Berg et al. [46] demonstrated that the sensitivity and specificity of combined mammography, ultrasound, and breast MRI was 100% and 65%, respectively. In contrast, the sensitivity of mammography only was 52%, while the specificity was 91%.

In recent years, the clinical application of preoperative breast MRI in patients with newly diagnosed breast cancer has seen rapid expansion as the modality becomes increasingly more available to clinicians [47]. Its use, however, remains controversial. No consensus has been reached concerning the utility of breast MRI in preoperative staging of all women with newly diagnosed breast cancer, and its benefit remains the topic of unremitting debate. The publication of the COMICE trial highlighted that the addition of contrast-enhanced MRI to conventional triple assessment was not associated with reduced reoperation in a large randomized cohort of women with primary breast cancer [48]. However, these findings were counter-balanced by evidence that preoperative MRI delineates patients with additional diseases that may be indistinguishable on conventional imaging [49,50,51,52]. 

In 2010, the European Society of Breast Cancer Specialists (EUSOMA) working group, consisting of a panel of twenty-three experts from a spectrum of disciplines including epidemiology, genetics, oncology, radiology, radiation oncology, and surgery took steps to evaluate the evidence and establish a consensus opinion for breast MRI indications [44]. Recommendations were detailed on appropriate use for; MRI for staging before treatment planning; high-risk screening; response evaluation following neoadjuvant treatment and patients with an occult primary on conventional methods, amongst others. This position paper highlighted the need for breast cancer specialists to adhere to the recommendations with patient outcomes as the primary endpoint. Despite this, the breadth of conflicting literature regarding the use of MRI for breast cancer management demonstrates that consensus opinions are still somewhat divergent.

## 6. Microwave Breast Imaging (MBI)

The shortcomings of mammography, such as relatively low sensitivity, radiation exposure, and uncomfortable breast compression, have resulted in research into alternative methods of breast imaging. Microwave breast imaging (MBI) has been highlighted as an exciting method for the detection of diseased breast tissue, offering a potential non-ionizing, non-compressive approach to breast cancer diagnosis [53] and monitoring the effect of neoadjuvant chemotherapy [54]. The potential of microwave imaging for clinical application has been researched over the last forty years [55]. Despite considerable efforts, only limited progress has been made in translating this potential into a clinically useful modality, as demonstrated by review articles from 1982 and 2016 describing microwave imaging as a “promising imaging modality” [56,57]. 

Microwave imaging uses electromagnetic radiation with frequencies between 0.3 and 9.0 GHz to deduce the dielectric properties (permittivity and conductivity) or contrast within a set volume, termed the imaging domain. To acquire data, the imaging volume is illuminated with electromagnetic radiation which propagates through the imaging domain and is scattered by dielectric contrasts in the imaging domain. The backscattered data is then recorded and used for image formation. A variety of systems exist, some which reconstruct a 3D volume, and others reconstruct repeated coronal slices of the breast to reduce the imaging algorithm complexity and to accelerate image reformatting [58]. Quantitative or qualitative image reconstruction algorithms are then used to estimate the dielectric contrast within the imaging domain. Qualitative algorithms, such as radar-based imaging, detect regions within the imaging domain where the dielectric properties fluctuate from the surrounding tissue, while quantitative algorithms, such as tomography, establish the actual dielectric properties for specific foci from the propagation paths through the imaging domain.

Microwave imaging is mostly analogous to sonography, where microwave energy is used instead of high-frequency sound, and scattering is based on the dielectric properties of tissues instead of the acoustic impedance. However, many differences exist between the two modalities which warrant further investigation:

- Respective beam widths of the transducer and the antennas;
- The relative dielectric properties and acoustic properties of the tissues of interest which is not well-studied;
- Importance of contact between the skin and the transducer and the antenna.

The dielectric properties, and hence the propagation, reflection, and attenuation of the electromagnetic waves in the microwave frequency range is sensitive to water, and accordingly, the water content in tissue plays a crucial factor in tissue characterization with microwave imaging [59]. Tissue water content has been shown to be higher in carcinogenesis due to a progressive increase in cell hydration induced by successive genetic and epigenetic changes [60]. Morphologically, cytoskeletal filaments are highly dynamic structures mainly involved in cell movements and proliferation. However, in the setting of an undifferentiated tumor cell, the cytoskeleton has a preponderance to be imperfect and dysfunctional due to a reduction in the quantity and organization of microtubules and microfilaments. This can result in the loss of normal cell structure and an altered cell membrane permeability. As a result, the tumor cells preserve more fluid than healthy cells, and this additional fluid alters the dielectric properties of the tissue [61].

Plasma constitutes 60% of blood content and is largely made up of water (92% by volume) [62]. In a tumor microenvironment, neoangiogenesis occurs to facilitate and nourish cell proliferation. This results in a network of new capillaries, and as the tumor increases in dimension, these networks can develop into small vessels that connect to major blood vessels, resulting in a significantly higher throughput of blood in the region. This increased volume of water in the tumor microenvironment is responsible for greater electromagnetic conductivity and permittivity. The dielectric contrast of the cancerous tissue against the surrounding healthy tissue results in increased scattering at the site of the tumor, thus enabling the detectability of the cancerous tissue with microwave imaging [63].

At least seven operational systems have been employed for numerous trials to investigate the clinical utility of MBI. These studies include healthy control volunteers or patients with benign breast disease or invasive carcinoma and are briefly summarized.

In 2000, the first of five clinical trials employing a tomography-based operational system developed at Dartmouth College (DC), USA, was described [64]. With this prototype, the breast is actively illuminated with a 16-element transceiving monopole antenna array in the 300–1000 MHz range. These trials ranged in size from 23 patients to 150 patients with and without breast disease and included a study investigating the role of the DC system in neoadjuvant chemotherapy monitoring [54,64,65,66,67]. In the cohort investigated undergoing neoadjuvant chemotherapy (*n* = 8), a positive correlation was identified between conductivity and 30-day response pathological response in all patients [54].

Outcomes from the Multistatic Array Processing for Radiowave Image Acquisition (MARIA^®^) systems, developed at the University of Bristol, UK, have been published since 2010, and report the largest outcomes from operational MBI systems. Six clinical studies have been reported describing the use of this radar-based system ranging in size from 86 patients [53] to 223 patients [68]. This system was designed with a 60-element antenna array system that consists of 60 wide-slot antenna elements positioned in a hemispherical arrangement. The antennas operate over a frequency range of 3 to 8 GHz in a cavity loaded slot arrangement. The most up-to-date system, the MARIA^®^5, was redesigned to improve the clinical utility of the system: with a reduced data acquisition time, immediate data processing to allow a DICOM compatible image reconstruction in less than 2 min. The sensitivity of this system is claimed to be as high as 86% for cancers in dense tissue [68]. This system has been made commercially available by Micrima Ltd. and has been deployed in several symptomatic breast units in the North Bristol Trust as part of the undergoing clinical evaluation of the system [66]. At least seven further operational systems had been employed for numerous trials to investigate the clinical utility of MBI, each reporting favorable outcomes [68]. 

In 2013 the first clinical report was published assessing a system developed by the University of Calgary, Canada, called the Tissue Sensing Adaptive Radar (TSAR) system. In this pilot study, the findings from eight patients were reported. The images showed responses consistent with the known clinical history in five patients, while various degrees of ambiguity surrounded the remaining three patients due to patient complexity and technical issues during data acquisition [69]. While the number of patients included in the initial trial was small, further clinical trials are planned with a new generation system [70,71].

In 2017, a group from the Southern University of Science and Technology, China, described the early results of a radar-based system which has been employed in Phase I clinical trials of 11 female Asian patients, aged between 22 and 47, with varying levels of hyperplasia [72]. Further phases of trials are scheduled using this system. As Asian women have denser breast parenchyma than their European counterparts, these results will be essential in assessing the clinical utility of microwave imaging in dense breasts. Also, in 2017, a group from Hiroshima University, Japan, described early results of a handheld, radar-based system [73]. This study included five patients, all of whom had either IDC or DCIS. Images were reconstructed without insight into the tumor location or clinical history of the patient. Despite this, the group demonstrated that the pathology could be located and that their findings were consistent with the clinical history of the patient.

In 2016, a wearable device system was described by Porter et al. at McGill University, Quebec, Canada [74]. In this study, 13 healthy control patients were evaluated. Imaging using this device was found to be comfortable, and measurements were reproducible, although sources of variability, such as patient positioning, were highlighted. As this study was confined to healthy control volunteers, the need for further clinical evaluation of the system is clear.

A system developed at Shizuoka University, Japan, has been described [75]. This ultra-wideband radar system had between 6 and 30 antennae to accommodate various breast sizes. This study, however, was limited to two participants, and as such, the clinical utility of this system will require further investigation.

Early reports from an ongoing clinical study at the Clinical Research Facility of Galway University Hospital (CRFG) have been described [76,77]. The Wavelia system is a low-power electromagnetic wave operational system consisting of two subsystems, both performing a non-compressive, non-invasive breast examination. The first subsystem, the optical breast contour detection (OBCD) subsystem, serves to gather information to help increase the accuracy of the subsequent microwave breast imaging subsystem. The OBCD subsystem consists of a 3D optical camera placed at 15–20 cm below an examination table and determines the volume and boundary outline of the subject breast. An azimuthal scan of the 3D camera allows the contour of the breast to be reconstructed, and the breast volume deduced. The second subsystem, microwave breast imaging, is performed on a separate examination table, where a vertical scan of the pendulous breast is performed, and consecutive coronal sections obtained. Multistatic radar detection technology is employed to detect and localize Regions-Of-Interest (ROIs) of breast tissue, with significant dielectric contrast on each coronal slice of the scanned breast. The Wavelia microwave imaging system operates using 18 wideband Vivaldi-type antennas arranged in a circle in a horizontal plane outside the cylinder containing the coupling fluid. Preliminary results from this study were favorable, with initial MBI demonstrating unambiguous detectability of cancerous lesions, with clusters of significant radar target echoes in the region of known underlying carcinoma [77]. The first phase of this clinical investigation has been completed, consisting of patients with benign and malignant breast disease. The findings of this study will determine the potential and potential clinical utility of the Wavelia system.

## 7. Prospects for Microwave Breast Imaging

Despite its limitations, mammography remains the gold standard against which new imaging technologies are measured. The adjunct of sonography and, more recently, tomosynthesis into clinical practice have improved breast cancer detection, while the increasing use of breast MRI is having a favorable impact when used in the appropriate setting. Although technological advances have brought about improvements in imaging quality and developments in modalities, there remains a chasm between current and optimal breast cancer detection levels, with approximately 2.4–6% of patients still being identified with de novo stage-IV breast cancer at the time of initial diagnosis [78]. While mortality rates are declining, the incidence of this multifaceted disease continues to increase with survival dependent on the stage at diagnosis [5]. Microwave imaging is rapid and inexpensive and may offer an additional adjunct to mammography, or perhaps a stand-alone modality in the future. If the exciting preliminary results of several operating systems are substantiated, the non-ionizing, non-invasive, and painless characteristics of this modality will popularize its implementation in routine diagnostic breast care. While several study populations to date have been too small to determine clinical efficacy accurately, the favorable preliminary results from the larger trials such as the DC and MARIA^®^ have indicated that microwave imaging may have an integrated role in breast imaging in the near future [66,68]. From a clinical perspective, further clinical trials will require a significantly higher number of patients to validate the true potential of the systems. Ideally, these trials should have a variety of breast pathology to represent the heterogeneity of breast disease, including invasive ductal carcinoma, invasive lobular carcinoma, fibroadenoma, and cystic breast disease. 

As we approach an age where the translation of microwave breast imaging systems into the clinical setting has become a reality, it will be imperative that the findings from microwave breast imaging studies are interrogated by breast radiologists with an aim to determine consensus opinions about multiple factors. These will include issues like reporting methods and terminology for describing findings, image presentation for the radiologist (i.e., 3D representation or standard anatomical planes such as coronal, axial and sagittal), imaging features (e.g., clusters/ foci of signal) and limits of signal that may suggest malignancy or benign disease. Furthermore, it will be crucial that the overseeing physician (radiologist), will play a role in developing standard operating procedures to limit any psychological or physical side-effects of MBI on a patient, considering the sensitivity of cancer imaging.

## Figures and Tables

**Table 1:** Randomized trials in mammography screening.

Trial	Country
Greater New York Health Insurance Plan (HIP) [14]	United States
Two-County Trial (TCT) [15]	Sweden
Malmo Mammography Screening Trial (MMST) [16]	Sweden
18-year follow-up meta-analysis of Swedish RCTs [17]	Sweden
Goteborg trial [18]	Sweden
National Breast Screening Study-1 (NBSS-1) [19]	Canada
National Breast Screening Study-2 (NBSS-2) [20]	Canada
Trial on women 40 years old at entry [21]	United Kingdom
